# RhoA/rho-kinase, nitric oxide and inflammatory response in LIMA during OPCABG with isoflurane preconditioning

**DOI:** 10.1186/s13019-019-0835-9

**Published:** 2019-01-25

**Authors:** Liang Zhang, Cheng-Bin Wang, Bo Li, Duo-Mao Lin, Jun Ma

**Affiliations:** 1 Department of Anesthesiology, Beijing Anzhen Hospital, Capital Medical University-Beijing Institute of Heart Lung and Blood Vessel Diseases, No.2 Anzhen Road, Chaoyang District, Beijing, 100029 China; 2 Department of Cardiac Surgery, Beijing Anzhen Hospital, Capital Medical University-Beijing Institute of Heart Lung and Blood Vessel Diseases, No.2 Anzhen Road, Chaoyang District, Beijing, 100029 China

**Keywords:** Isoflurane, Left internal mammary artery, OPCABG

## Abstract

**Background:**

Grafting vessel with LIMA to the left anterior descending coronary artery plays a most important role in the long-term prognosis of OPCABG surgery. The aim of this study was to compare the effects of isoflurane preconditioning on miRs and mRNAs levels in the left internal mammary arterie (LIMA) graft with propofol in patients undergoing off-pump coronary artery bypass surgery (OPCABG).

**Methods:**

Patients were randomly assigned to receive either propofol (*n* = 15), or interrupted isoflurane (*n* = 15). In group P, propofol administration was continued at 3–5 mg/kg/h intravenous injection for the duration of surgery. Five minutes prior to incision, patients of the isoflurane group (group Iso) received 2 cycles of 1 MAC isoflurane.

**Results:**

miR-221 were significantly lower in group Iso (*P* < 0 .05). E-selectin mRNA, RhoA mRNA and ROK mRNA were significantly lower at specimens of LIMA in group Iso compared with those in group P patients (*P* < 0 .05). The expression of NOS3 mRNA was significantly higher in group Iso patients (*P* < 0 .05).

**Conclusion:**

Our findings provide some insight that prior interrupted isoflurane administration could regulate miR-221, and downstream effectors (mRNAs) and resulted in actual attenuation of inflammation and spasm of LIMA in patients undergoing OPCABG surgery.

**Trial registration:**

NCT No. (ClinicalTrials.gov): NCT02678650; Registration date: January 23, 2016.

## Background

Coronary artery bypass grafting (CABG) surgery is the standard treatment for patients with 3-vessel coronary artery disease. Reports [1, 2] have showed that autologous arteries rather than veins as bypass graft provide superior long-term outcomes. Moreover, using of LIMA to graft the diseased left anterior descending coronary artery has become the standard method for almost all CABG surgery [3]. However, graft spasm, especially in LIMA with an incidence of at least 0.43% in all CABG surgery [4], and graft stenosis still cause the problem in patients undergoing CABG surgery.

miRs are small non–coding RNAs that regulate the expression of target protein–coding genes by promoting the degradation or suppressing the translation of target mRNAs. Evidence shows that miRs act as key regulators for endothelial biology and function [5].

Recent studies have also shown that volatile anesthetics can influence miRs expression profiles in the liver and cardiomyocytes [6, 7], suggesting that isoflurane preconditioning may influence miRs in LIMA as well. In addition, the regulatory effects of anesthetics on proinflammatory cytokines and NO/eNOS are demonstrated by experimental data [8]. Thus, in this study, we hypothesize that isoflurane-mediated preconditioning could protect LIMA via down-regulation of miRs. To test this, we compare the effects of isoflurane on miRs and mRNAs levels in LIMA graft with propofol in patients undergoing OPCABG surgery.

## Methods

### Patients

We performed a randomized-, prospective-, controlled clinical study. This investigation conforms to the principles outlined in the Declaration of Helsinki after receiving the Medical Ethics Committee approval at Beijing Anzhen Hospital, Capital Medical University. Written informed consent was obtained from all patients prior to inclusion. This study is registered in the ClinicalTrials.gov database (ID: NCT02678650), Clinical trial date of registration was 23/01/2016, and was performed between January 2016 and December 2016 at Beijing Anzhen Hospital.

Patients with American Society of Anesthesiologists (ASA) scores between class II and III having coronary artery revascularization by OPCAB surgery for 3-vessel disease were included. Exclusion criteria were angina during the previous 72 h, unstable angina, acute myocardial infarction, ejection fraction lower than 40%, the need for inotropic agents or an intra-aortic balloon pump preoperatively, congestive heart failure, emergency procedures, former CABG surgery, concurrent valve repair, severe systemic diseases involving the renal and hepatic systems, respiratory disease (forced vital capacity less than 50% of predicted values), or theophylline therapy.

### Anesthesia and surgery

Patients received 10 mg of morphine intramuscularly as premedication one hour prior to entering the operating room. Standard monitoring was achieved in all the patients. Anesthesia was induced with sufentanil (1 μg/kg), etomidate (0.3 mg/kg), cisatracurium (0.2 mg/kg) and maintained with hypnotics (propofol or isoflurane), cisatracurium, and opioid (sufentanil). Patient monitoring included continuous 5-lead electrocadiographic registration with ST-segment analysis, peripheral oxygen saturation by pulse oximetry, radial arterial blood pressure, central venous pressure, capnography, rectal temperature, and urine output. The radial artery catheter was connected to a monitor for pulse contour analysis (MostCare system, Vygon-Vytech, Padova, Italy) and the resulting signal processed for determination of hemodynamic variables. Depth of anesthesia was determined with bispectral index (BIS XPTM, Aspect Medical Systems, Newton, MA, USA) and aimed at BIS values between 40 and 50 during surgery. According to the study protocol, body-position changes, vasoactive drugs (e.g., norepinephrine), and dopamine were used to keep mean arterial pressure (MAP) above 70 mmHg. The nasopharyngeal temperature was held above 36.0 °C. All OPCABG procedures were performed by the same surgical team.

### Intervention

According to computer-generated randomization, patients were randomly assigned to receive either propofol (group P, *n* = 15), or interrupted isoflurane (group Iso, *n* = 15). In group P, propofol administration was continued at 3–4 mg/kg/h for the duration of surgery. In group Iso, 5 min prior to incision, began to isoflurane wash-in / wash-out operation: isoflurane administration was interrupted for 10 min, by washed out to achieve a MAC value below 0.2(with a high fresh gas flow 8 L.min^− 1^). Following the interruption, isoflurane to achieve 1 MAC end-tidal concentration as soon as possible (with a high fresh gas flow 6 L.min^− 1^), and repeated twice periods of 10 min. Discontinuation of isoflurane for at 15 min during the last wash out time. When isoflurane inhaled anesthetic, propofol was stopped infusion. If during this interruption the BIS value increased to > 50, 0.5 mg/kg propofol was administered repeatedly in boluses until the BIS value returned to < 50. The mean flow (MF) and pulsatility index (PI) of the LIMA graft were measured at 10 min after OPCABG when the ultrasonic coupling index (ACI) was ≥90% by an Ultrasonic Blood Flow Detector (Medi-stim, VeriQ 4122, Norway).

### Tissue sampling

At 1 h after isoflurane exposure (group P, about 100 min after incision), a segment (1 cm) of LIMA was taken from 30 subjects undergoing OPCABG surgery by sgurgeon. We excluded patients who received papaverine or high-dose vasoactive drugs before the LIMA graft. Before we obtained LIMA, surgeon did not apply any vasodilator such as papaverine. In all patients, LIMA was freed firstly and then the distal end of the internal mammary artery was severed. After we got the pedicled LIMA, LIMA was carefully dissected from their surrounding tissue, and then snap-frozen in liquid nitrogen and kept at − 80 °C until further analyses were carried out.

### Quantitative RT-PCR

Total RNA was extracted using the TRIzol reagent kit (BioTeke, China), and RNA purity, integrity and quantity were examined by nanodrop (NANO 2000, Thermo, USA). Purified RNA samples were performed on reverse transcribed cDNA using a reverse-transcription kit (BioTeke, China). Real-time PCR was performed with 2 μ L of diluted RT product in a Exicycler™ 96 (BIONEER, Korea) using SYBR Green PCR Supermix (Solarbio, China) according to the manufacturer’s instructions. U6 was used as reference for miRs and β-actin for mRNAs. Target expression was normalized to the expression of loading control for each sample and the difference between samples was calculated using the 2 ^- △△ CT^ method. The designed primers are listed in Table 1.

**Table 1:** List of the designed primers

miR or mRNA	Sequence(5′¬3′)
miR-221 F	GCGACCACCTGGCATACAAT
miR-221 R	GTGCAGGGTCCGAGGTATTC
miR-146 F	GCGAGGTGAGAACTGAATTCCA
miR-146 R	GTGCAGGGTCCGAGGTATTC
U6 F	CTCGCTTCGGCAGCACA
U6 R	AACGCTTCACGAATTTGCGT
NOS3 F	TGTTTGTCTGCGGCGATGT
NOS3 R	GGTGCGTATGCGGCTTGT
RhoA F	GTGCCCACAGTGTTTGAGA
RhoA R	ATCGGTATCTGGGTAGGAG
IKB-a F	ATGAAAGACGAGGAGTACGAG
IKB-a R	GCAGGTTGTTCTGGAAGTTGA
VCAM-1 F	ATTTGACAGGCTGGAGATAGAC
VCAM-1 R	CAGCCTGCCTTACTGTGGG
E-selectin F	AGAGGCAGCAGTGATACCC
E-selectin R	TGAGAAGCACCAAAGTGAGAG
ROK F	TGACTGAGTGCCCTGTGGA
ROK R	AACCCTGAAGCCTGTGATA
β-actin F	CTTAGTTGCGTTACACCCTTTCTTG
β-actin R	CTGTCACCTTCACCGTTCCAGTTT

### Western blot (WB)

Biopsies were kept stored at − 80 °C and prepared for analysis on the same day. To measure protein expression, total protein concentration was measured by the BCA Protein Assay Kit (Pierce, Rockford, IL, USA). 40 μg protein samples were loaded and separated on a 5–10% SDS-PAGE gradient gel, and electrically transferred onto a PVDF membrane (Millipore, Billerica, USA). PVDF membranes were blocked with 5% nonfat milk in Tris buffer solution containing 0.1% Tween-20 (TBST) for 1 h at room temperature, followed by incubation with primary antibody. After washing four times with TBST (5 min each, room temperature), the membranes were incubated for 1 h with fluorescent-labeled secondary antibodies (anti-Rabbit IgG at 1:5000). Then, the membranes were washed and proteins were visualized by using an enhanced peroxidase/luminal chemiluminescence reaction (ECL Western blotting detection reagent) (Wanleibio, China), and the final images were analyzed by densitometry using Gel-Pro-Analyzer software (Media Cybernetics, USA). All primary and secondary antibodies were purchased from Wanleibio Biology Ltd., Shenyang, China.

### Statistical analysis

Sample size calculation was based on the assumption that the SD of the means of the TnI plasma levels will be 1.5 ng/mL 4 h after surgery, a = 0.05, and β = 0.8. A 40% decrease with isoflurane preconditioning was assumed [9], the power analysis indicated that a minimum sample size of 10 patients was required for each group. In this study we enrolled 15 patients to improve the power of test.

Descriptive analysis was performed using mean ± SD for continuous variables and frequencies (percentages) for categorical data. For comparisons of categorical variables Chi-square analysis was used. Student’s t-test was used to compare the means of two samples. All of the collected data were stored electronically and analyzed using SPSS 17.0 software (SPSS Inc., Chicago, IL, USA). Kolmogorov–Smirnov and normal-quantile plots were used to determine whether the continuous variables were normally distributed. Graphics were produced by use of GraphPad Prism 5 (GraphPad Software, San Diego, CA).

## Results

### Patient characteristics

Major complications, in-hospital and 30-days mortality did not occur in this study. Table 2 summarizes characteristics and clinical parameters of the perioperative patient (*n* = 30) in the two study groups. There were no significant differences in terms of age, height, body weight, sex, diabetes mellitus (receive insulin therapy one week before surgery), hypertension, and LVEF, or in other parameters, such as MF, PI values, ventilation duration, ICU stay and hospital stay.

**Table 2:** Patient characteristics and perioperative parameters

	group Iso	group *P*	*p*-Value
Gender, male/female	12/3	10/5	0.539
Age (year)	57.5 ± 5.2	57.3 ± 8.2	0.217
Weight (kg)	74.9 ± 9.8	69.6 ± 13.1	0.430
Height (cm)	166.7 ± 7.2	164.6 ± 7.4	0.855
Diabetes mellitus	3	5	0.409
Hypertension	11	10	0.690
Left ventricular ejection fraction (%)	60.8 ± 7.6	63.1 ± 6.6	0.379
Ventilation (hours)	11.0 ± 2.9	12.4 ± 2.6	0.185
ICU stay (hours)	14.5 ± 2.5	16.9 ± 4.3	0.075
Hospital stay (days)	9.7 ± 1.1	9.8 ± 1.2	0.755
Number of bridge vessels	4.0 ± 0.6	3.8 ± 0.6	0.642
nitroglycerin prior to surgery	3	4	0.666
MF(l/min)	37.3 ± 12.3	31.3 ± 10.6	0.199
PI	2.2 ± 0.9	1.9 ± 0.6	0.146

ICU: intensive care unit

### Myocardial injury

**Fig. 1:**
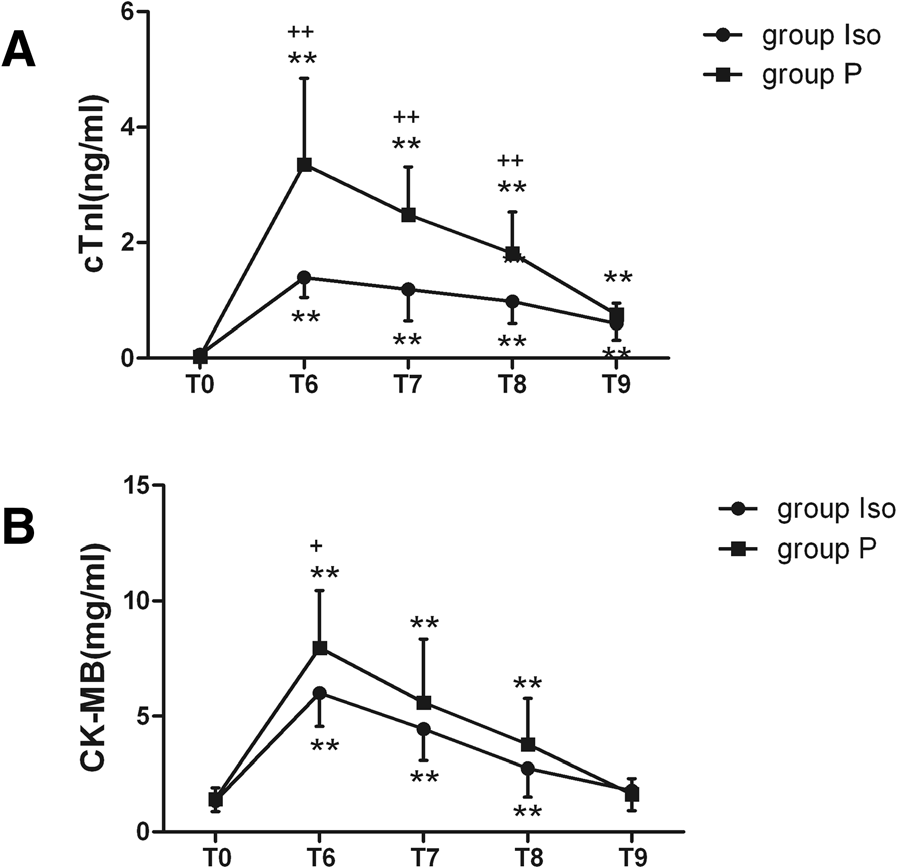
Postoperative release of serum cTnI and CK-MB. (**a**) Serum cTnI levels at different time points. (**b**) Serum CK-MB levels at different time points. * *P* < 0.05, ** *P* < 0.01, compare with T0; + *P* < 0.05, ++ *P* < 0.01, between the groups. T0, 5 min prior to incision; T1, 4 h after surgery; T2, 12 h after surgery; T3, 24 h after surgery; T4, 48 h after surgery

The two groups had similar baseline preoperative serum cTnI and CK-MB values. cTnI and CK-MB values were higher in both groups in the periods after surgery and peaked at the 4th hour after surgery in both groups. At 4 h after surgery, the cTnI and CK-MB values in the patients in the Iso group were lower compared to the P group (*P* < 0.05). (Fig. 1).

### Quantitative RT-PCR for miRs

**Fig. 2:**
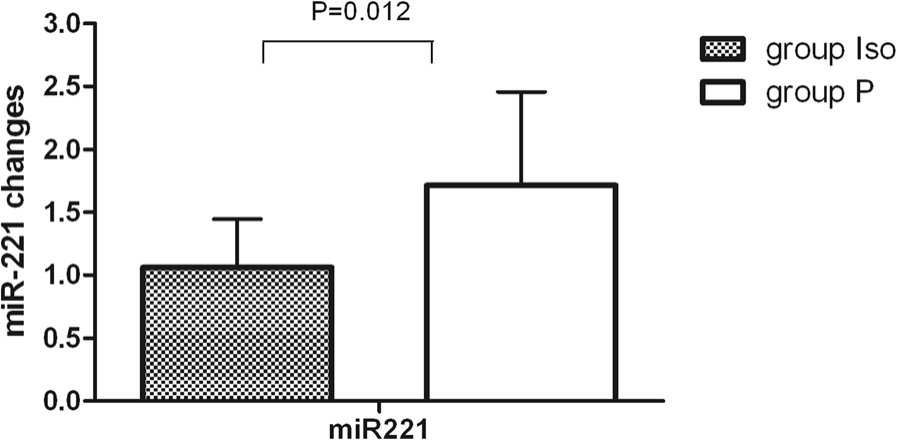
Expression change of microRNAs (miRs) in LIMA during OPCABG. Expression change of miR-221 in both groups

We found that compared with the absence of isoflurane preconditioning, isoflurane pretreatment significantly down-regulated miR-221 expression in LIMA at 1 h after isoflurane exposure(*P* < 0.05). Further, miR-221 acts as key regulators for endothelial biology and function [10–12]. (Fig. 2).

### Expression of NO-associated genes

**Fig. 3:**
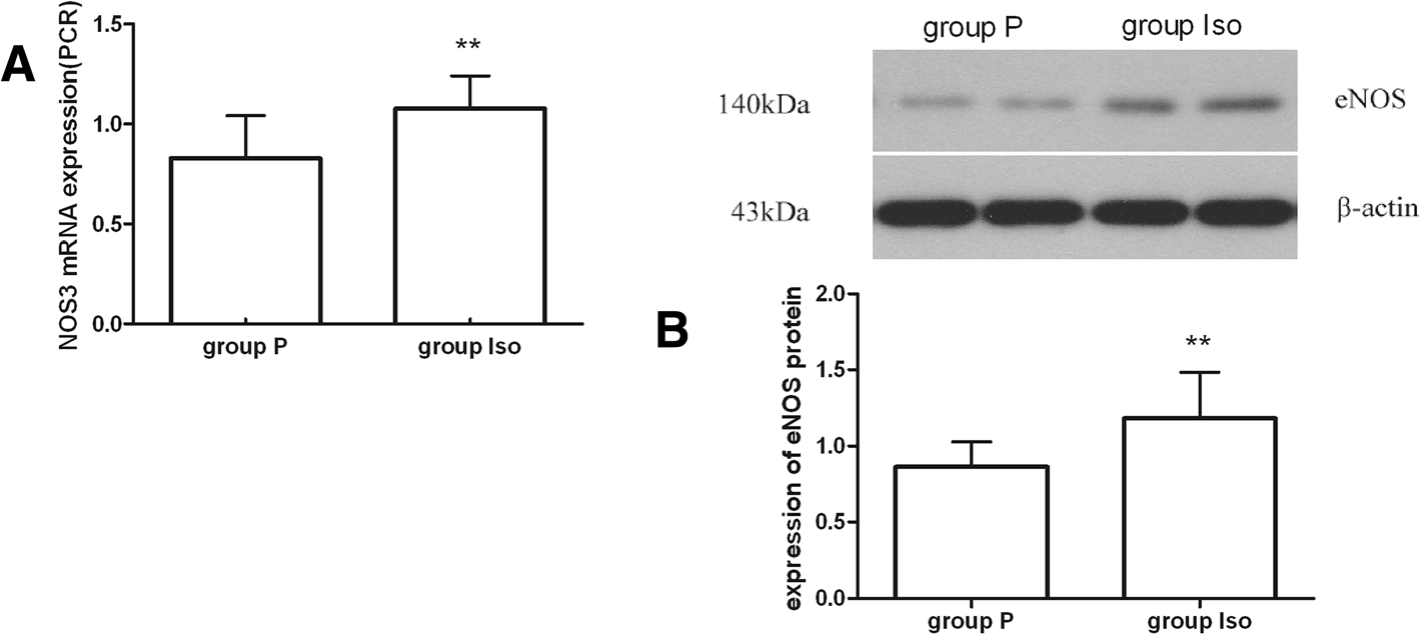
Expression of NO-associated genes. (**a**) Expression change of NOS3 mRNA. (**b**) Expression change of eNOS protein

NO, synthesized by endothelial NO synthase (eNOS) which is a putative target gene of miR-221, is very important for vascular function [13]. Our data indicated that NOS3 mRNA and eNOS protein increased significantly at 1 h after isoflurane preconditioning(*P* < 0.05) and suggested isoflurane pretreatment regulation of eNOS translational efficiency. (Fig. 3).

### Expression of inflammatory-associated genes

**Fig. 4:**
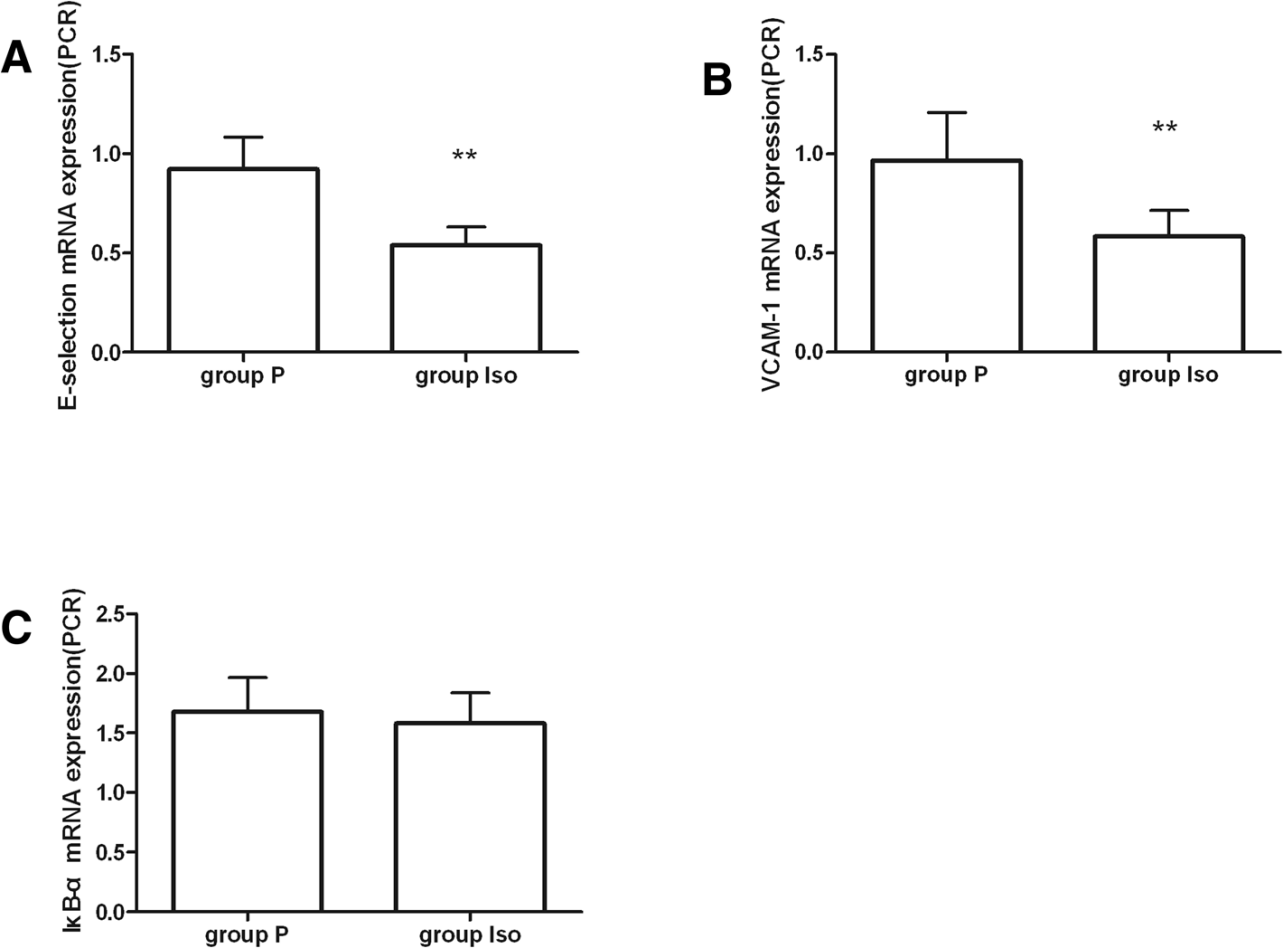
Expression of inflammatory-associated genes. (**a, b**) Expression change of E-selectin and VCAM-1 mRNA. (**c**) Expression change of IκB-a mRNA

Nuclear factor-kappa B (NF-κB) is considered to be another putative target gene of miR-221 [14]. We found that the expression of E-selectin mRNA and VCAM-1 mRNA levels decreaced after isoflurane exposure(*P* < 0.05). However, IκB-a mRNA was not significantly changed in group Iso compared with group P(*P* = 0.316). (Fig. 4).

### Expression of spasm-associated genes

**Fig. 5:**
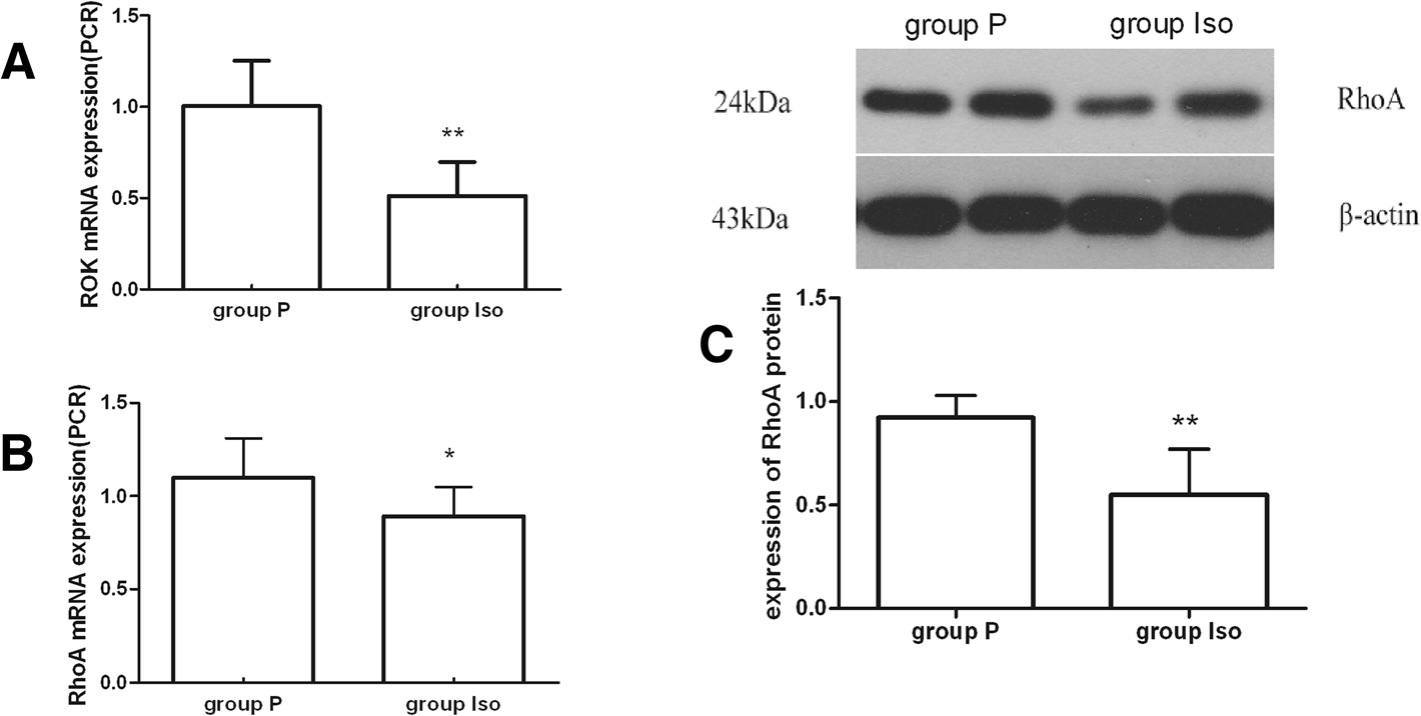
Expression of spasm-associated genes. (**a, b**) Expression change of RhoA and ROK mRNA. (**c**) Expression change of RhoA protein

The pathway of RhoA/Rho kinase (RhoA/ROK) is a major cellular target for regulating vasoconstriction [15]. RhoA and ROK were studied at the mRNA and protein levels using quantitative RT-PCR and Western blotting. The results showed that group P had a significantly higher level of mRNA expression of RhoA and ROK than of group Iso, and RhoA protein was also expressed higher in group P(*P* < 0.05). (Fig. 5).

## Discussion

The detailed mechanisms of the influence of anesthetic agents on LIMA are still unknown. The primary findings of the present study were that isoflurane preconditioning during the OPCAB surgery for 3-vessel disease attenuated myocardial injury, and reduced inflammatory response and vasospasm in LIMA. More importantly, results also indicated that miR-221 expression were decreased in group Iso and their common target genes changed accordingly in LIMA. A growing body of evidence shows an organ protective effect by volatile anesthetics, such as isoflurane and sevoflurane [16, 17]. In addition, miRs are known to be involved in the protective effect of volatile anesthetics [18–20]. In accordance with our findings, the protective effect of LIMA of isoflurane preconditioning could be at least in part attributed to miR-221.

miR-221 plays a key important role in vascular biology through its effects on vascular smoothmuscle cells and endothelial cells. For example, miR-221 is found to be changed in vascular disease, such as atherosclerosis, coronary artery disease, and post-angioplasty restenosis [21–23]. In addition, evidence showed [14] that miR-221 inhibited NO production and abolished the inhibitory effect of adiponectin on E-selectin activation in human umbilical vein endothelial cells, which was in line with our study. Olson and colleagues [20] demonstrated that miR-21 is acting to protect cardiomyocytes after isoflurane exposure. However, our study demonstrated that miR21 was not changed between groups but miR-221 and were down-regulated after isoflurane exposure in LIMA, and LIMA and cardiomyocytes tissue differences may be a major reason for the difference between the two results.

Taken together, eNOS and E-selectin seem to be the common targets of miR-221 to regulate inflammatory. Although the long-term patency of LIMA graft principally depends on the severity of the native coronary artery stenosis and the quality of the distal vascular bed, the formation of atherosclerosis [24] which is caused by inflammation and reduced NO production in the vascular endothelium also plays a key role in the restenosis of LIMA. eNOS which has been proposed as a major factor involved in inflammatory process [25] is expressed mainly in endothelial cells and involved in regulating vascular function. Meanwhile, E-selectin is essential for the expression of inflammatory genes and endothelium pathology [26]. In this study, by quantitative RT-PCR and WB, our results indicated that eNOS was up-regulated and E-selectin mRNA was down-regulated in LIMA in group Iso. Ge et al. [27] also reported that NO functions as both a trigger and a mediator of cardioprotection produced by iso post-conditioning. Smul et al. [28] showed that early preconditioning with desflurane produces a marked reduction in infarct size in an in vivo myocardial infarction rabbit model by desflurane-induced NOS activation. In addition, Huang et al. [29] reported that myocardial tissue eNOS protein expression in a joint isoflurane and propofol anaesthesia was significantly higher than those in the control group. Although regulatory effects of anesthetics on NO/eNOS are demonstrated by experimental data [30], the detailed regulatory mechanisms are still not fully understood. Here, by our study results, we hypothesized that isoflurane exposure inhibits the increase in miR-221 expression in LIMA, which attenuates inflammatory in LIMA. The exactly cause-effect relationship in our hypothesis was not fully supported by our findings. Further detailed regulatory mechanisms studies are thus needed.

Our another finding was that the expression of RhoA/ROK was down-regulated after isoflurane preconditioning. RhoA/ROK-kinase signaling pathways which is a major cellular target for regulating Ca2+ sensitivity of agonist-induced contraction [31] is a most important contraction mechanisms of artery smooth muscle cell, the activation of RhoA leads to stimulation of ROK that can favor myosin light chain (MLC) phosphorylation, actin-myosin interaction and cell contraction [32]. However, whether miRs regulate expression of RhoA/ROK in arterial grafts induced by isoflurane exposure remain unknown. Yang et al. [33] demonstrated that Iso inhibit KCl-induced PI3K-C2α-participation, Rho kinase-mediated MLC phosphorylation, and vasoconstriction in rat aortic smooth muscle. Here, our data indicated that isoflurane preconditioning supressed the expression of RhoA/ROK and activated endothelial eNOS, resulting in vasodilation of LIMA, so we demonstrated that isoflurane preconditioning may be very useful for reperfusion of ischemic myocardium after revascularization for left anterior descending coronary artery. However, cellular mechanistic experiments may be needed in the futuer.

The limitations of the current study are discussed as follows. First, in this study, experimental time under anesthesia was only 1 h after isoflurane exposure. There is a need to verify time-dependent changes of miRs and mRNAs expression. Second, the tissue samples were only taken from LIMA. Given the difference among great saphenous vein, radial artery and LIMA, our results in LIMA may be different with other grafting vessels. However, grafting vessel with LIMA to the left anterior descending coronary artery plays a most important role in the long-term prognosis of OPCABG surgery. Our results at least provide some insight into how isoflurane preconditioning reduces inflammation and relieves vasospasm in LIMA via miR-related pathways.

## Conclusions

In conclusion, the results obtained in our study revealed that isoflurane preconditioning applied during OPCABG surgery indicated cardioprotective effects, which can be partly explained by its ability to inhibit inflammatory responses and vasoconstriction in LIMA grafting. Further, isoflurane preconditioning altered the expression of miR-221, which may help better understand the underlying mechanisms of isoflurane preconditioning.

## Abbreviations

ASA: American Society of Anesthesiologists
CK-MB: Creatinine kinase-MB
cTnI: Cardiac troponin I
eNOS: Endothelial NO synthase
LIMA: Left internal mammary arterie
MAC: Minimum alveolar concentration
miRs: MicroRNAs
NF-κB: Nuclear factor-kappa B
OPCABG: Off-pump coronary artery bypass surgery
VCAM-1: Vascular cell adhesion molecule 1
